# The Neuroconnective Endophenotype, A New Approach Toward Typing Functional Neurological Disorder: A Case-Control Study

**DOI:** 10.1192/j.eurpsy.2025.1025

**Published:** 2025-08-26

**Authors:** M. Martínez García, A. Bulbena-Vilarrasa, L. Pintor Pérez, M. Camara, N. Arbelo-Cabrera, A. Bulbena-Cabré, V. Pérez-Sola, C. Baeza-Velasco

**Affiliations:** 1 Psychiatry, Mental Health Institute, Hospital del Mar; 2 Psychiatry and Forensic Medicine, Universitat Autònoma de Barcelona, Barcelona; 3 Centro de Investigación Biomédica en Red de Salud Mental, Instituto de Salud Carlos III, Madrid; 4 Psychiatry and Psychology, Consultation Liaison Psychiatry Unit, Hospital Clinic de Barcelona, Institute of Neuroscience; 5 Medicine, University of Barcelona; 6 August Pi i Sunyer Institute of Biomedical Research; 7 Consultation Liaison Psychiatry Unit, Hospital Clinic de Barcelona, Institute of Neuroscience, Barcelona, Spain; 8 Psychiatry, Icahn School of Medicine at Mount Sinai, New York, United States; 9 Mental Health Institute, Hospital del Mar; 10 Facultad de Ciencias de la Salud y de la Vida, Universitat Pompeu Fabra, Barcelona, Spain; 11 Laboratoire de Psychopathologie et Processus de Santé, Université Paris Cite, Paris; 12 Department of Emergency Psychiatry and Acute Care, University of Montpellier; 13 Centre National de la Recherche Scientifique, Inserm, Institute of Functional Genomics, Montpellier, France

## Abstract

**Introduction:**

Functional neurological disorder (FND) is a core neuropsychiatric condition that includes both physical and mental symptoms. Recently, a validated clinical phenotype termed neuroconnective endophenotype (NEP), which includes several physical and psychological characteristics together with joint hypermobility (hypermobility spectrum disorders), was found at a significantly higher frequency among patients with anxiety (Bulbena A *et al.* Adv Psychosom Med 2015; 34:143–157).

**Objectives:**

The purpose of the present study was to examine the presence of the NEP among patients with FND.

**Methods:**

A multicenter case-control study was conducted comprising 27 FND patients and 27 healthy control participants (matched by sex and age) ages 13 to 58 years. Eight questionnaires were administered. Proportional differences were examined with Student’s t tests, one-way analyses of variance, and chi-square tests.

**Results:**

Differences between FND patients and control participants were observed. As presented on tables 1 and 2, FND patients had higher sensory sensitivity, increased prevalence of hypermobility features (including relevant physical signs and symptoms), greater frequency of polarized behaviors and an increase in the characteristics and sensations typical of anxiety. FND patients also presented a greater number of comorbidities, both psychiatric (generalized anxiety disorder or panic attacks (63%); phobias, including social anxiety, agoraphobia, and specific phobias (52%); and depression (44%)) and physical (migraine (59%), irritable bowel syndrome (48%), drug intolerances (44%), dark sclerae (41%), and chronic fatigue syndrome (41%)). Particularly striking was the presence of the hypermobility spectrum in more than 75% of FND patients compared with 15% among control participants (Table 3).

**Image 1:**

**Figure:**
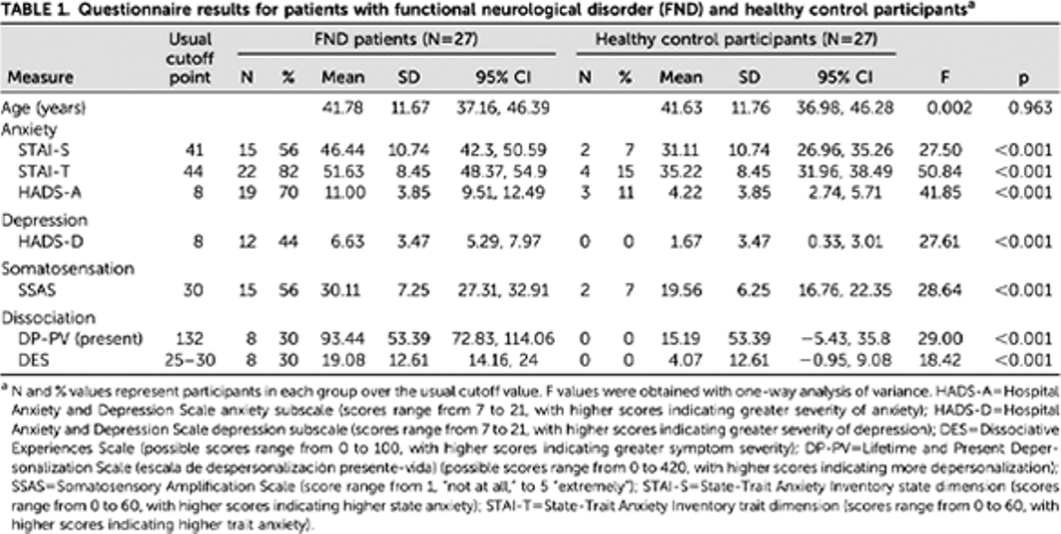

**Image 2:**

**Figure:**
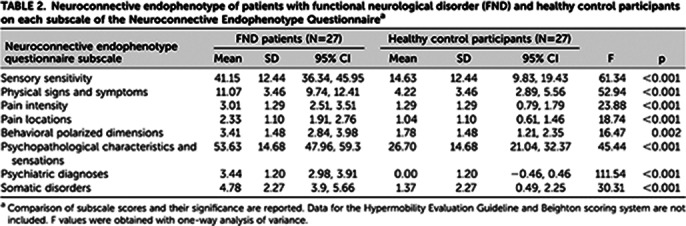

**Image 3:**

**Figure:**
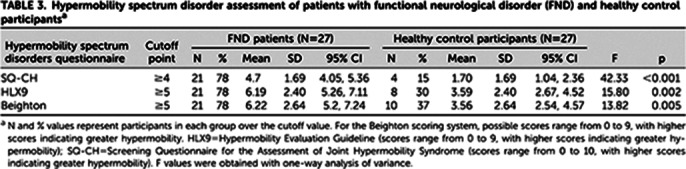

**Conclusions:**

FND patients presented higher scores in all five dimensions included in the NEP. Thus, this phenotype, solidifying the original association between anxiety and the hypermobility spectrum, could help to identify an FND subtype when evaluating and managing FND patients, because it provides a new global view of patients’ physical and mental symptoms. Limitations in this study included small sample size, possible selection and recall biases and lack of data on socioeconomic factors and education.

**Disclosure of Interest:**

M. Martínez García: None Declared, A. Bulbena-Vilarrasa: None Declared, L. Pintor Pérez: None Declared, M. Camara: None Declared, N. Arbelo-Cabrera: None Declared, A. Bulbena-Cabré: None Declared, V. Pérez-Sola Grant / Research support from: AB-Biotics, AstraZeneca, Bristol-Myers Squibb, Centro de Investigación Biomédica en Red de Salud Mental, FIS-ISCiii, Janssen Cilag, Lundbeck, Otsuka, Servier and Medtronic, Consultant of: AB-Biotics, AstraZeneca, Bristol-Myers Squibb, Centro de Investigación Biomédica en Red de Salud Mental, FIS-ISCiii, Janssen Cilag, Lundbeck, Otsuka, Servier and Medtronic, C. Baeza-Velasco: None Declared

